# First report of *Bursaphelenchus fungivorus* (Nematoda: Aphelenchida) in Italy and an overview of nematodes associated with *Crocus sativus* L.

**DOI:** 10.21307/jofnem-2020-023

**Published:** 2020-03-30

**Authors:** Giulia Torrini, Agostino Strangi, Stefania Simoncini, Maria Luppino, Pio Federico Roversi, Leonardo Marianelli

**Affiliations:** 1 CREA Research Center for Plant Protection and Certification, Via di Lanciola 12/A, Cascine del Riccio, 50125 Firenze, Italy; 2 Regione Lazio – Direzione Regionale Agricoltura, Promozione della Filiera e della Cultura del Cibo, Caccia e Pesca – Area Servizio Fitosanitario Regionale Innovazione in Agricoltura – Via del Serafico, 107, 00142 Roma, Italy

**Keywords:** Central Italy, Molecular biology, Morphology, Mycophagus nematode, Saffron

## Abstract

Saffron is one of the most precious spices in the world. It is derived from the dried stigmas of the *Crocus sativus* L. flowers. This plant is triploid sterile and propagated by corms. As a subterranean organ, the corm can come into contact with different nematodes. In this contribution *Bursaphelenchus fungivorus* was reported for the first time in Italy. It was found associated with *C. sativus* corms and characterized based on morphological and morphometrical characteristics for this species. The identification was confirmed using molecular analyses. Moreover, a review of nematodes associated with *C. sativus* worldwide is also provided.

Saffron is one of the most expensive spices worldwide and it is obtained from the flower stigmas of *Crocus sativus* L., which belongs to the family of Iridaceae and to the genus *Crocus*, which includes about 90 species distributed mainly in the Mediterranean and southwestern Asia (Fernández et al., 2011; Dar et al., 2017). The world’s total annual saffron production is estimated at 205 tons, and over 80% of this harvest originates from Iran (Mahdikhani and Alvani, 2013; Kafi et al., 2018). Outside Europe, saffron is cultivated mainly in Kashmir, China, Afghanistan, Azerbaijan, Turkey and Morocco. Europe has always played a significant role in the international production and commercialization of saffron with Spain, Greece and Italy being the main producers of this spice (Kafi et al., 2018). The most extensive cultures of saffron in Italy are located in Abruzzo, Sardinia and Tuscany, where there is growing interest about guaranteeing and defending the quality of this product with Protected Designations of Origin (Cusano et al., 2018).

Particularly in the last century, saffron cultivation areas and production amounts have decreased worldwide, largely due to the manual harvesting of stigmas, high production costs and low yields of the spice. In fact, 450,000 hand-picked delicate red strands from 150,000 flowers are required to obtain 1 kg of saffron (Grilli-Caiola and Canini, 2010; Kafi et al., 2018).

*Crocus sativus* is a triploid sterile plant, propagated by corms. As a subterranean organ, the corm can come into contact with different organisms such as fungi, bacteria, viruses and nematodes. Most information concerning nematodes associated with saffron, refers to nematodes collected in the soil around the roots of *Crocus sativus* (Fotedar and Handoo, 1977; Mahdikhani and Alvani, 2013; Sheikh et al., 2014; Cirujeda et al., 2016; Alvani et al., 2017; Hassan and Ahangar, 2018).

In this study, *Bursaphelenchus fungivorus* (Franklin and Hooper, 1962) is reported for the first time in Italy, and, in particular, associated with *C. sativus* corms. Morphometrical and molecular characterization of the nematode is given. In addition, a review of nematodes associated with plants of *C. sativus* worldwide is provided.

## Material and methods

### Collecting corms and nematodes extraction

In September 2018, 20 corms of *Crocus sativus* were collected by the Plant Protection Service of Lazio Region on the farm “Arte Zafferano” located in Terracina (Latina, Lazio, Central Italy), in the karst area of Campo Soriano. The farm is characterized by labyrinths of calcareous rocks, dotted by small drained plots where saffron is cultivated. This survey was conducted to certify that these plants were free of plant-parasitic nematodes, such as *Ditylenchus destructor* (Thorne, 1945) and *Ditylenchus dipsaci* (Kühn, 1857; Filipjev, 1936), for their exportation of the corms to Guatemala (Central America).

Corms were put in polyethylene bags and then brought to the laboratory of Nematology at CREA-DC in Florence (Italy). These materials were kept in the refrigerator at about +4°C until they were processed for analysis. A mat of parallel fibers that shroud the corms (corm tunic) was removed and the naked corms were washed with sterilized water and then cut into 2 mm slices. Then the two materials were separately processed using the modified Baermann funnel method (Schindler, 1961). Isolated nematodes were observed with a dissection microscope to determine their presence and then under a light microscope for morphological identification at the genus or species level.

Specimens of *Bursaphelenchus* (Fuchs, 1937) were transferred to a 9 cm diameter Petri dish with 2% malt extract agar and *Botrytis cinerea* lawn in order to obtain a culture. After a two week incubation at 26°C, nematodes were collected for morphobiometrical and molecular studies.

### Morphological identification

*Bursaphelenchus* specimens were collected from the Petri dish, washed in sterilized water and heat-killed in warm water at 65°C; then they were fixed in triethanol-amine-formalin (Courtney et al., 1955), processed in glycerin by a modification of the glycerin-ethanol series of Seinhorst (1959) rapid method and finally, permanently mounted in anhydrous glycerin on glass microscope slides.

In total, 10 females and 10 males were photographed and measured. Photographs were taken with a Leica DM2000 light microscope using a Leica MC170 HD digital camera (Leica, Heerbrugg, Switzerland). Measurements were performed with the LEICA Application Suite (LAS) Version 4.9.0. Morphological and molecular characteristics were compared with the original description (Franklin and Hooper, 1962).

### Molecular analysis

Two specimens of nematodes were individually put in 0.2 ml tube containing 50.0 μl InstaGene Matrix (BioRad), 1.5% SDS and 2.5 μl Proteinase K 20.0 μg/μl. The samples were incubated at 55.0°C for 3 hr, Proteinase K was inactivated by heating at 96.0°C for 10 min and DNA was recovered with alcoholic precipitation adding 100.0 μl of cold absolute ethanol. Pellets were air-dried and resuspended in 20.0 μl of double distilled water. The amplification of Internal Transcribed Spacer locus (ITS) was performed using conditions described in Burgermeister et al. (2005). The PCR products were sequenced at the Centro di Servizi per le Biotecnologie di Interesse Agrario Chimico e Industriale, University of Florence, Italy. Species identification was obtained through two phylogenetic trees (obtained with Neighbor Joining and Maximum likelihood algorithms) based on ITS locus and focused on *fungivorous*-group starting from sequences of this locus mined from GeneBank (Table 1). Alignments were computed with a local alignment algorithm (implemented in Kalign at EBI website) and the poorly aligned regions were removed with Gblocks v. 9.1b. The choice of appropriate substitution matrix for the data set was evaluated using Jmodeltest2 v. 2.1.10 considering AICc, BI criteria and DT method. Trees were computed using MEGA 7 software, choosing GTR +G +I as nucleotide substitution matrix and 1,000 bootstrap replicates.

**Table 1.** Species list and sequences used in this work annotated with their GeneBank Accession numbers.

*Bursaphelenchus* species	Strain, isolate or clone	GeneBank Accession
*B. arthuri*	Ne19/04	AM157742
*B. arthuri*	–	EU783918
*B. arthuroides*	32468	HQ599189
*B. braaschae*	14190	GQ845407
*B. cocophilus*	S5	KT156782
*B. cocophilus*	S8	KT156783
*B. fungivorus*	BfungPt1 Clone 1	KF241745
*B. fungivorus*	BfungPt1 Clone 2	KF241746
*B. fungivorus*	BfungPt1 Clone 3	KF241747
*B. fungivorus*	GD	HQ402559
*B. fungivorus*	GT2018	MK372853
*B. fungivorus*	Ne 26/96	AM179516
*B. parathailandae*	00349	JN377723
*B. thailandae*	HK	DQ497183
*B. thailandae*	Ne7b/03	AM157746
*B. thailandae*	RCA	KP644768
*B. thailandae*	UN	KP644769
*B. willibaldi*	Ne 16/05	AM180512

## Results

### Morphological identification

**Figure 1:**
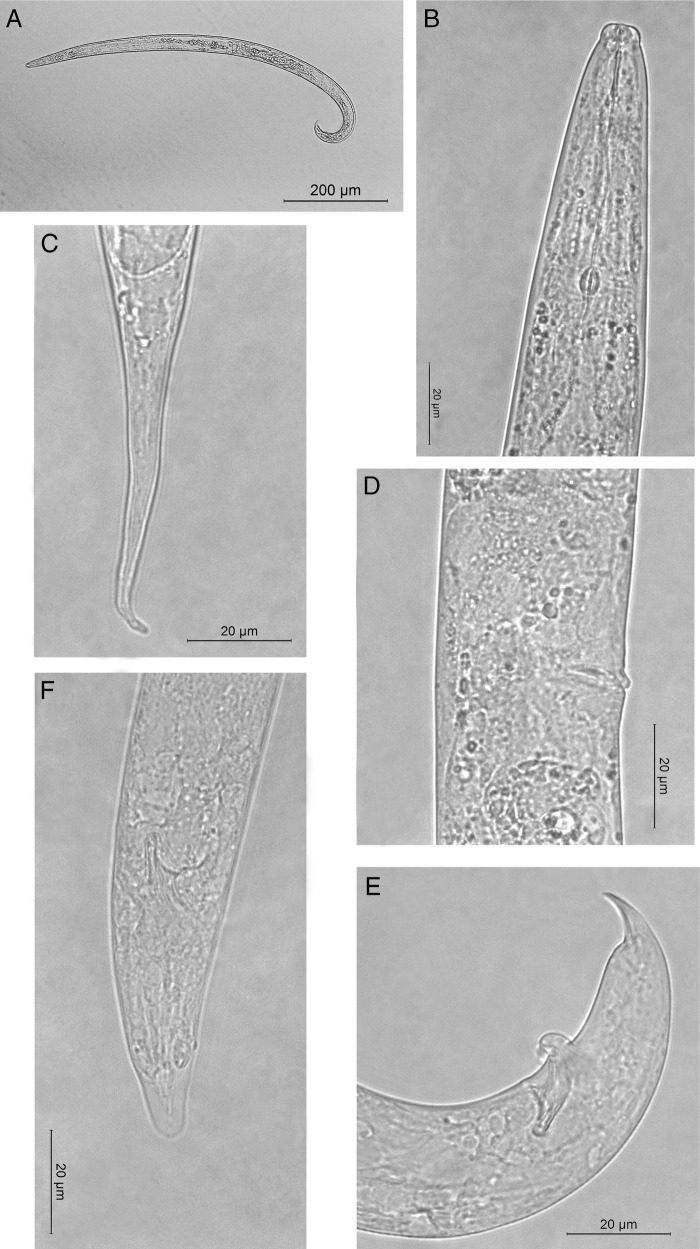
Light micrographs of *Bursaphelenchus fungivorus.* A: Male total body; B: Female anterior region; C: Female tail; D: Vulval region; E: Lateral view of male tail; F: Ventral view of male tail (Scale bar A = 100 μm; B-F = 20 μm).

No specimens belonging to the *Ditylenchus* genus were isolated from these samples, while some nematodes belonging to the *Bursaphelenchus* genus were extracted, but only from naked corms. Specimens presented the main morphological characteristics of *Bursaphelenchus fungivorus*: four lateral incisures, cephalic region offset by a constriction, stylet with weakly developed basal knobs; well-developed median bulb. Females present elongated conical tail with a rounded tip and ventrally bent. Vulva is without a flap in lateral and ventral views; both anterior and posterior vulval lips slightly protruding. In the males, tails are ventrally curved with compact spicules without cucullus (Fig. 1). The morphology of the Italian population of *B. fungivorus* (Table 2) agrees with the original description and with this species found in other countries (Braasch, 2001; Arias et al., 2005; Fonseca et al., 2014).

**Table 2.** Morphometrics of *Bursaphelenchus fungivorus*.

Character	Male	Female
n	10	10
L	686.7 ± 31.9	735.28 ± 38.4
	(645.2 – 742.0)	(700.0 – 834.8)
a	28.3 ± 1.9	26.5 ± 2.0
	(25.3 – 30.9)	(23.3 – 30.3)
b	10.2 ± 0.8	10.5 ± 0.5
	(9.2 – 11.8)	(10.1 – 11.7)
c	20.5 ± 1.0	12.4 ± 0.5
	(19.1 – 22.1)	(11.8 – 13.1)
c'	2.2 ± 0.2	5.4 ± 0.4
	(1.9 – 2.5)	(4.8 – 6.3)
T or V	64.1 ± 4.9	73.1 ± 0.4
	(57.5 – 72.8)	(72.4 – 73.7)
Max. body diam.	24.4 ± 1.8	27.9 ± 2.1
	(22.4 – 27.9)	(24.2 – 31.7)
Stylet	13.7 ± 0.8	13.4 ± 1.5
	(12.6 – 14.9)	(11.3 – 16.2)
Median bulb length	17.4 ± 2.0	19.6 ± 1.7
	(14.3 – 20.3)	(17.1 – 22.0)
Median bulb diam.	11.9 ± 1.1	14.9 ± 1.8
	(10.8 – 13.3)	(13.0 – 19.3)
Tail length	33.7 ± 2.3	59.2 ± 2.6
	(30.5 – 38.4)	(56.7 – 65.0)
Cloacal or anal body diam.	15.5 ± 1.1	10.9 ± 0.5
	(13.8 – 17.1)	(10.2 – 11.8)
Spicule length*	15.3 ± 1.1	–
	(13.7 – 17.5)	

Notes: All measurements are in μm: mean ± SD (range). *Curved along arc from bottom of capitulum depression to distal end.

### Molecular analysis

**Figure 2:**
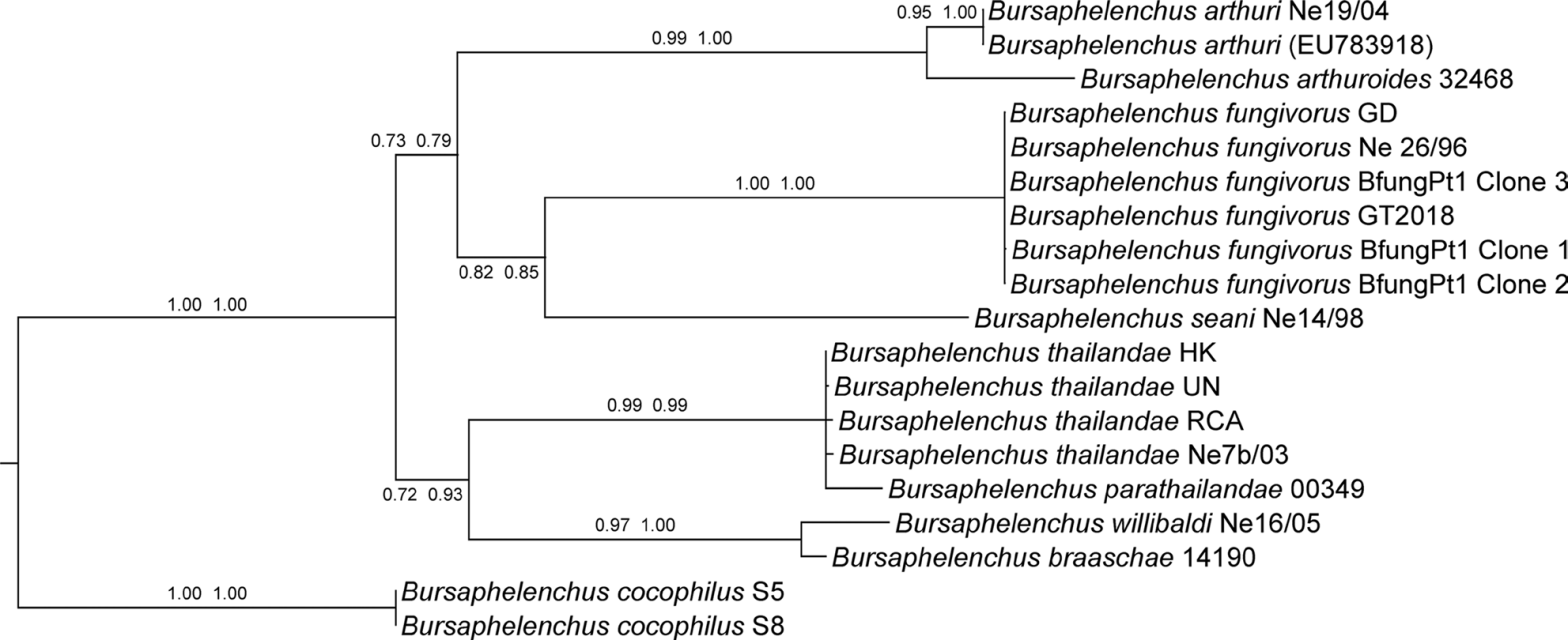
Phylogenetic relationships of species belonging to *fungivorus*-group based on ITS locus. The tree was inferred using GTR + I + G as nucleotide substitution model, tested with 1,000 bootstrap replicates and *Bursaphelenchus cocophilus* was chosen as outgroup species. Nodes were annotated with NJ and ML bootstrap values, respectively.

The two nematodes vouchered had the same sequence in the ITS locus with a length of 969 bp, this sequence was submitted in GeneBank with accession number MK372853. Both trees obtained with maximum likelihood and neighbor joining methods share the same topology. The resulting tree (Fig. 2) showed a well-supported subdivision of *fungivorus*-group into two main clades, the first includes *B. fungivorus*, *Bursaphelenchus seani* (Giblin and Kaya, 1983), *Bursaphelenchus arthuri* (Burgermeister et al., 2005) and *Bursaphelenchus arthuroides* (Gu et al., 2012) while the second *Bursaphelenchus thailandae* (Braasch and Braasch-Bidasak, 2002), *Bursaphelenchus parathailandae* (Gu et al., 2012), *Bursaphelenchus willibaldi* (Schönfeld et al., 2006) and *Bursaphelenchus braaschae* (Gu and Wang, 2010). This finding is according to Kanzaki et al. (2016) where the same result was obtained starting from SSU and LSU combined sequences. Based on these results, we identified the nematodes as *Bursaphelenchus fungivours*s voucher GT2018.

## Discussion

The international trading of *Crocus* corms is considered a possible pathway of nematode introductions in new pest-free areas, where this crop is important for the local economy. For this reason, some plant-parasitic nematodes are regulated in trade in many countries of the world.

Collecting pest reports and notifications of nematodes isolated from *C. sativus*, a review of nematodes is presented in Table 3. A wide diversity of genera and species were associated with this crop; to be noted that the greatest number were extracted from soil. Most isolated nematodes have been reported in Asian countries, while only one species, *Ditylenchus dipsaci*, has been isolated in Africa. Spain, Greece and Italy are the only European countries where nematodes have been found associated with *C. sativus*.

**Table 3.** Occurrence of nematodes associated with *Crocus sativus* L. in different geographic location.

Feeding habitat	Genus	Species	Matrix of extraction	Country	References
Bacteria	*Acrobeles*	*Acrobeles* spp.	Soil	Spain	Cirujeda et al. (2016)
	*Acrobeloides*	*Acrobeloides* spp.	Soil, Corm	Spain	Cirujeda et al. (2016)
	*Boleodorus*	*B. thylactus*	Soil, Root	Iran	Saeedizadeh et al. (2018)
	*Cephalobus*	*Cephalobus* spp.	Soil	Spain	Cirujeda et al. (2016)
	*Rhabditis*	*Rhabditis* spp.	Soil	Spain	Cirujeda et al. (2016)
Fungi	*Aphelenchoides*	*A. asterocaudatus*	Soil, Root	Iran	Mahdikhani and Alvani (2013), Saeedizadeh et al. (2018)
		*A. blastophthorus*	Soil, Corm, Leaves	Spain	Cirujeda et al. (2016)
		*A. centralis*	Soil, Root	Iran	Saeedizadeh et al. (2018)
		*A. curiolis*	Soil	Iran	Mahdikhani and Alvani (2013)
	*Bursaphelenchus*	*B. fungivorus*	Corm	Italy	Present study
	*Ditylenchus*	*D. myceliophagus*	Soil, Root	Iran	Mahdikhani and Alvani (2013), Saeedizadeh et al. (2018)
Fungi/Plant	*Aphelenchoides*	*A. besseyi*	Soil	Iran	Mahdikhani and Alvani (2013)
		*A. subtenuis*	Soil, Corm	Greece	Koliopanos and Kalyviotis-Gazelas (1979), Decker (1989)
	*Aphelenchus*	*A. avenae*	Soil, Corm	Spain, India, Iran	Zaki and Mantoo (2008), Cirujeda et al. (2016), Saeedizadeh et al. (2018)
Omnivorus	*Ecumenicus*	*Ecumenicus* spp.	Soil	Spain	Cirujeda et al. (2016)
Plant	*Aerotylenchus*	*A. safroni*	Soil	Kashmir	Fotedar and Handoo (1977)
	*Amplimerlinius*	*A. globigerus*	Soil, Root	Iran	Alvani et al. (2017), Saeedizadeh et al. (2018)
		*A. icarus*	Soil	Iran	Mahdikhani and Alvani (2013)
		*A. macrurus*	Soil	Iran	Mahdikhani and Alvani (2013)
		*A. socialis*	Soil	Iran	Mahdikhani and Alvani (2013)
	*Criconemoides*	*C. deccipiens*	Soil	Iran	Mahdikhani and Alvani (2013)
	*Ditylenchus*	*D. dipsaci*	Soil, Corm	Azerbaijan, Iran, Marocco	Kasimova and Atakishieva (1980), Radouni (1985), Ait-Oubahou and El-Otmani (1999), Mahdikhani and Alvani (2013)
	*Filenchus*	*F. afghanicus*	Soil, Root	Iran	Saeedizadeh et al. (2018)
		*F. hamatus*	Soil	Iran	Mahdikhani and Alvani (2013)
		*F. pratensis*	Soil	Iran	Mahdikhani and Alvani (2013)
		*F. vulgaris*	Soil, Root	Iran	Saeedizadeh et al. (2018)
	*Geocenamus*	*G. squamatus*	Soil	Iran	Mahdikhani and Alvani (2013)
		*G. tenuidens*	Soil	Iran	Mahdikhani and Alvani (2013)
	*Helicotylenchus*	*Helicotylenchus* spp.	Soil	Kashmir, Spain	Sheikh et al. (2014), Cirujeda et al. (2016)
		*H. chishtii*	Soil, Root	Kashmir	Sheikh et al. (2014)
		*H. crassatus*	Soil	Iran	Mahdikhani and Alvani (2013)
		*H. exallus*	Soil	Iran	Mahdikhani and Alvani (2013)
		*H. pseudorobustus*	Soil, Root	Iran	Saeedizadeh et al. (2018)
		*H. vulgaris*	Corm	India	Zaki and Mantoo (2008)
	*Hemicriconemoides*	*Hemicriconemoides* spp.	Corm	India	Zaki and Mantoo (2008)
	*Hirschmaniella*	*Hirschmaniella* spp.	Soil, Root	Kashmir	Sheikh et al. (2014)
	*Meloidogyne*	*M. brevicauda*	Root	Azerbaijan	Kasimova and Atakishieva (1980)
	*Merlinius*	*M. bavaricus*	Soil	Iran	Mahdikhani and Alvani (2013)
		*M. brevidens*	Soil	Iran	Mahdikhani and Alvani (2013), Alvani et al. (2017)
		*M. graminicola*	Soil	Iran	Mahdikhani and Alvani (2013)
		*M. microdorus*	Soil, Root	Iran	Mahdikhani and Alvani (2013), Saeedizadeh et al. (2018)
		*M. nanus*	Soil	Iran	Mahdikhani and Alvani (2013)
		*M. pseudobavaricus*	Soil	Iran	Mahdikhani and Alvani (2013)
	*Nagelus*	*N. camelliae*	Soil, Root	Iran	Saeedizadeh et al. (2018)
		*N. hexagramus*	Soil, Root	Iran	Saeedizadeh et al. (2018)
	*Paratylenchus*	*P. coronatus*	Soil, Root	Iran	Mahdikhani and Alvani (2013), Saeedizadeh et al. (2018)
		*P. similis*	Soil, Root	Iran	Saeedizadeh et al. (2018)
	*Pratylenchoides*	*P. alkani*	Soil	Iran	Alvani et al. (2017)
	*Pratylenchus*	*Pratylenchus* spp.	Soil, Root	Kashmir	Sheikh et al. (2014)
		*P. coffeae*	Soil	Iran	Mahdikhani and Alvani (2013)
		*P. crenatus*	Different part	Azerbaijan	Kasimova and Atakishieva (1980)
		*P. loosi*	Soil	Iran	Mahdikhani and Alvani (2013)
		*P. neglectus*	Soil, Root	Iran	Saeedizadeh et al. (2018)
		*P. penetrans*	Soil	Azerbaijan, Iran	Kasimova and Atakishieva (1980), Mahdikhani and Alvani (2013)
		*P. pratensis*	Different part	Azerbaijan	Kasimova and Atakishieva (1980)
		*P. thornei*	Soil, Root, Corm	Azerbaijan, Iran, India	Kasimova and Atakishieva (1980), Zaki and Mantoo (2008), Mahdikhani and Alvani (2013), Saeedizadeh et al. (2018)
	*Psilenchus*	*Psilenchus* spp.	Soil	Kashmir, Spain	Sheikh et al. (2014), Cirujeda et al. (2016)
		*P. elegans*	Soil	Iran	Mahdikhani and Alvani (2013)
		*P. hilarulus*	Soil, Root	Iran	Alvani et al. (2015), Saeedizadeh et al. (2018)
		*P. minor*	Soil	Iran	Mahdikhani and Alvani (2013)
	*Rotylenchus*	*Rotylenchus* spp.	Soil	Spain	Cirujeda et al. (2016)
	*Scutylenchus*	*S. rugosus*	Soil	Iran	Alvani et al. (2017)
		*S. tartuensis*	Soil	Iran	Alvani et al. (2017)
	*Tylenchorhynchus*	*Tylenchorhynchus* spp.	Corm	India	Zaki and Mantoo (2008)
		*T. brassicae*	Soil	Iran	Mahdikhani and Alvani (2013)
	*Tylenchus*	*Tylenchus* spp.	Soil, Root, Corm	Kashmir, Spain, India	Zaki and Mantoo (2008), Sheikh et al. (2014), Cirujeda et al. (2016)
		*T. arcuates*	Soil, Root	India	Hassan and Ahangar (2018)
		*T. kashmirensis*	Soil	India	Mahajan (1973)
		*T. parvus*	Soil	Iran	Mahdikhani and Alvani (2013)
	*Xiphinema*	*Xiphinema* spp.	Corm	India	Zaki and Mantoo (2008)

The source of food is fundamental to trophic interactions. In Table 3, nematodes with different feeding habitats are illustrated; in particular, plant feeders were the most abundant, but the pathogenic role of most of the species remains unclear.

This investigation was conducted in order to certify that these corms were free from plant-parasitic nematodes, a prerequisite for the exportation of *C. sativus* to Guatemala (Decreto N° 36-98 Ley de Sanidad Vegetal y Animal and Acuerdo Gubernativo N° 745-99 Reglamento de la Ley de Sanidad Vegetal y Animal, faculta al Ministerio de Agricultura, Ganadería y Alimentacíon). Plant-parasitic nematodes directly related to the *Crocus* corms have not been isolated during routine checks for *Crocus* corms exportation in non-EU countries, but numerous specimens of *Bursaphelenchus fungivorus* have been isolated and observed.

This species was first found in rotting *Gardenia* sp. buds infected by the fungus *Botrytis cinerea* in the UK (Franklin and Hooper, 1962). It was later found in Germany in a growing medium containing bark for Pelargonium plants. Both findings were located in greenhouses (Braasch et al., 1999). It was also detected in coniferous bark imported from the Czech Republic and Russia to Germany (Braasch et al., 2002). *Bursaphelenchus fungivorus* was also reported on *Pinus* spp. and associated with *Orthotomicus erosus* in Spain (Arias et al., 2004, 2005); and with *Pinus pinaster* bark in Portugal (Fonseca et al., 2014).

*Bursaphelenchus fungivorus*, as most *Bursaphelenchus* species, is a fungal feeding nematode. It is attracted to most of the fungus species, on which it can feed and reproduce (Townshend, 1964). Mycophagous nematodes can reduce or even stop the growth of fungi causing hyphal shrinkage (Riffle, 1971) and the application of this kind of nematodes for the control of some fungi harmful to crops has been described in several studies (e.g. Ishibashi and Choi, 1991; Lagerlöf et al., 2011).

Between the root-inhabiting fungi reported as hosts of *B. fungivorus* there are: *Alternaria solani*, *Botrytis cinerea*, *Fusarium culmorum*, *Fusarium oxysporum*, *Fusarium solani*, *Ophiobolus graminis*, *Pythium debaryanum*, *Pythium ultimum*, *Rhizoctonia solani*, *Trichoderma viride* and *Verticillim albo-atrum* (Townshend, 1964). Some of these have been reported as pathogens of saffron (Ahrazem et al., 2010), one of the most destructive diseases in *C. sativus* cultivation is *Fusarium oxysporum* because it causes severe yield losses (Cappelli, 1994).

*Bursaphelenchus fungivorus* is the most reported species among the nematode of the *funivorus*-group. The host range of the nematodes in this particular group is relatively wide with respect to the other *Bursaphelenchus* groups because the species have been found associated not only with coniferous dead or dying trees or insect vectors, but also in other type of trees (*Bursaphelenchus kiyoharai* (Kanzaki et al., 2011), *Bursaphelenchus maxbassiensis* (Massey, 1971), *Bursaphelenchus penai* (Kanzaki et al., 2014a), *Bursaphelenchus sycophilus* (Kanzaki et al., 2014b) and *B. willibaldi*), in the plant tissues of herbaceous crops (*B. fungivorus* and *Bursaphelenchus hunti* (Steiner, 1935; Giblin and Kaya, 1983), in soil (*Bursaphelenchus gonzalezi* (Loof, 1964) and *B. seani*) and in peat moss (*Bursaphelenchus rockyi* (Xu et al., 2018; Steiner, 1935; Loof, 1964; Massey, 1971; Mahajan, 1973; Fotedar and Handoo, 1977; Giblin and Kaya, 1983; Schönfeld et al., 2006; Kanzaki et al., 2011; Kanzaki et al., 2014a, 2014b; Xu et al., 2018). Since many *Bursaphelenchus* of the *fungivorus*-group have been found in subterranean organs of plants or directly in the soil, the nematodes of this group are likely to have a predisposition to underground life and are not closely related to the wood of the trees.

In conclusion, in this work, a new species of Italian nematode fauna was isolated, in particular, associated with *C. sativus* plants. Further research about the role of *B. fungivorus* as the control agent against pathogenic fungi on corms of *C. sativus* will have to be performed.
